# Editorial: Revisiting seed and soil: A new approach to target hibernating dormant tumor cells

**DOI:** 10.3389/fonc.2023.1126924

**Published:** 2023-01-30

**Authors:** Angélica Santiago-Gómez, Dalit Barkan, Ann F. Chambers

**Affiliations:** ^1^ Manchester Breast Centre, Division of Cancer Sciences, University of Manchester, Manchester, United Kingdom; ^2^ Molecular Oncology Programme, Spanish National Cancer Research Centre (CNIO), Madrid, Spain; ^3^ Department of Human Biology and Medical Sciences, University of Haifa, Haifa, Israel; ^4^ Departments of Oncology, Medical Biophysics, and Pathology & Laboratory Medicine, University of Western Ontario, London, ON, Canada

**Keywords:** dormancy, minimal residual disease, metastasis, tumour microenvironment, circulating tumour cells, cancer recurrence

Over hundred and thirty years have passed since Stephen Paget proposed his ‘Seed and Soil’ hypothesis about metastatic spreading (1). Since then, we have gained a better insight into the complexities of this multistep dispersal, but we are yet to fully comprehend the interactions governing the metastatic ecosystems to enable us to clinically prevent disease recurrence and, ultimately, cancer-related deaths.

Despite the high inefficiency of the metastatic process (2), cancer patients often suffer late recurrences following five to thirty years of undetectable disease (3, 4). This clinical observation is due to the presence of disseminated tumour cells (DTCs) that escape early from the primary tumour and spread to distant organs. Once there, DTCs may initially lie in a dormant or hibernating state to later reawaken, resulting in incurable metastatic outgrowths (5, 6). Dormant DTCs persist in a non-proliferative but reversible arrest, and consequently are resistant to conventional therapies directed towards rapidly dividing cancer cells. Moreover, they adapt to the metastatic niche they reside in, evading the immune system and reactivating tumour-initiating abilities when the opportunity arises (7, Weidenfeld and Barkan) (see (7) for concept definitions to avoid terminology misconceptions).

Therefore, metastatic dormancy represents a major clinical problem, as well as a novel window of opportunity to hamper metastatic relapse by interfering with the dormant cancer cell life cycle [key steps to control dormancy in (7)].

In this Research Topic, we revisited Paget’s hypothesis focusing on the dormant phase of metastatic progression. This 10-article collection provides an overview on recent advances in the dormancy field, including original research on organ-specific mechanisms driving the reawakening of DTCs; and comprehensive and exhaustive reviews about the microenvironmental regulation of dormancy and reawakening, cutting-edge technologies to study interactions with the metastatic microenvironment and innovative therapeutic strategies to clinically monitor and target this undetectable stage of metastatic disease (**Figure 1**).

**Figure 1:**
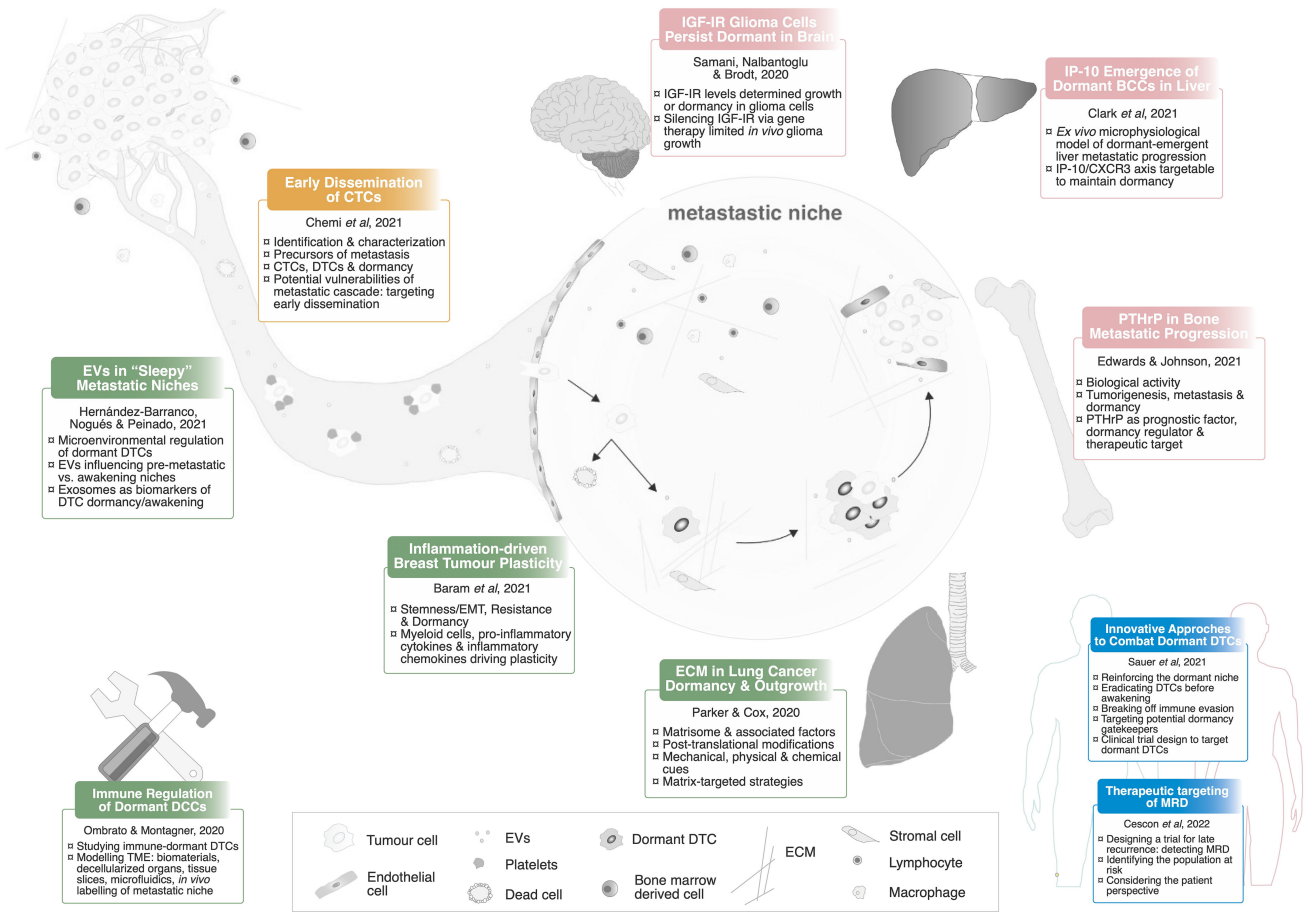
Revisiting the Seed and Soil hypothesis. All the articles included in the Research Topic are summarised in this schematic representation. CTCs, Circulating Tumour Cells; EVs, Extracellular Vesicles; DTCs, Disseminated Tumour Cells; EMT, Epithelial-to-Mesenchymal Transition; TME, Tumour Microenvironment; ECM, Extracellular Matrix; MRD, Minimal Residual Disease.

## Early dissemination

The journey of tumour cells with metastatic potential begins with their dissemination through the bloodstream and/or the lymphatic system. These traveller precursors of metastases, known as circulating tumour cells (CTCs), are extensively discussed by Chemi et al., who highlight their heterogeneity and suggest their potential utility as non-invasive biomarkers (liquid biopsy) to track minimal residual disease. Although initial spreading follows blood-flow patterns and vascular architecture (8) and few CTCs extravasate into secondary organs (9), Chemi et al. suggest that CTC molecular profiling could predict organotropism to a specific metastatic site.

## Organ-specific awakening of dormant DTCs

Most evidence we have gathered over years of research in the field shows intrinsic properties of the seeds in regulating dormancy at specific secondary sites. Molecular players such as TGFβ/BMP, p38/MAPK, NR2F1, uPAR, β1-integrins, IL-1β, among others, have been linked to dormancy or reawakening of metastatic cells in specific organs and cancer types (10–14). However, not all is black and white. In bone, Edwards and Johnson discuss in a comprehensive perspective that molecules such as PTHrP play opposing roles at different stages of disease progression and metastasis. In addition, there are two original research articles that highlight organ-specific molecular mechanisms involved in reawakening. Samani et al. show that downregulation of IGFR-I receptor limited glioma growth, promoting a dormant phenotype; Clark et al. use an *ex vivo* all-human liver microphysiological model to find that IP-10 promotes dormancy exit in the liver metastatic niche.

## Microenvironmental regulation of metastatic dormancy

Although Paget’s static notion drew attention to the importance of the surroundings (the soil), his hypothesis lacks evolving adaptation that occur in the metastatic ecosystem. The plasticity of the soil, either the tumour microenvironment (TME) or specific metastatic niches, is essential to understand the intricacies of metastatic progression, from primary tumour escape and intravasation, through CTC survival and extravasation at secondary sites, homing and DTCs survival to eventual colonisation.

Seeds and soils are not hermetic compartments, they reciprocally interact with and modulate each other. In fact, the primary tumour can even influence secondary organs to prepare the “congenial soil” or pre-metastatic niche *via* secreted factors and shed extracellular vesicles (EVs), preceding the arrival of the seeds (15). Hernández-Barranco et al. emphasise the need to unravel the communication mechanisms between DTCs and their metastatic niche, suggesting that secreted EVs could mediate this microenvironmental crosstalk to regulate dormancy. The authors also discuss the participation of EVs in the awakening of dormant metastatic cells as well as their potential utility as biomarkers to monitor minimal residual disease, suggesting a step forward for the liquid biopsy field.

Besides interacting with stromal cells, DTCs also display bidirectional communication with another essential structural element of the surroundings, the extracellular matrix (ECM). This three-dimensional network, consisting of macromolecules (such as proteins, proteoglycans, glycoproteins, cytokines and growth factors) modulates its own remodelling during the metastatic process (16–18). In fact, some ECM proteins such as tenascin C, periostin, type-I and type-XII collagen, among others, promote metastatic colonisation in different organs (19–22), whereas recent studies report that other ECM proteins such as thrombospondin-1, fibronectin, laminin-211 and type-III collagen sustain metastatic dormancy (23–26). Here, Parker and Cox exhaustively review the role of the ECM and associated factors in the regulation of tumour dormancy and metastatic outgrowth using the lung extracellular matrix as an example. The authors highlight that developing organ-specific ECM targeting strategies could reduce lung metastatic burden in lung and other solid cancers.

Recent advances in immunotherapy highlighted the role of the immune system in targeting cancer cells and therefore preventing metastasis. Although dormant metastatic cells evade both the innate and adaptive immunity (27) *via* intrinsic downregulation of activating receptors (28, 29), some immune populations such as NK cells contribute to sustaining a dormant phenotype at secondary sites (30). More details about the immunoregulatory control of survival and outgrowth of dormant DTCs can be found in Ombrato and Montagner. Another interesting aspect is the inflammatory microenvironment and its contribution to disease progression and metastasis (27). Baram et al. reviews recent findings on how inflammation (both driven by associated myeloid cells and other factors of the TME) influences tumour cell plasticity, focusing on the regulation of three areas: stemness and EMT, resistance to therapies and dormancy.

## Experimental model systems

However, this complex communication occurring within the metastatic ecosystem requires the development of cutting-edge techniques to dissect specific niche interactions and to study dormant DTCs, aiming for the discovery of more specific targets to prevent recurrence. Recent technological advances for studying interactions in the metastatic TME *in vitro* and *in vivo* are discussed by Ombrato and Montagner. The detailed list includes the use of biomaterials, decellularized organs, tissue slices, microfluidics and niche-labelling techniques.

## Therapeutic targeting of dormant metastatic cells

Once metastatic relapse occurs, cancer patients have very limited treatment options, and those few available choices usually rely on primary tumour features. Without doubt, metastatic dormancy provides a new window of opportunity to prevent relapse (10, 11, 31). But can we impact the clinical management of metastasis? And, most importantly, can we translate the lessons learnt at the bench into clinical practice? Sauer et al. and Cescon et al. give us clinical perspectives about potential strategies to target dormant DTCs and monitoring of patients with no evidence of disease. Interestingly, Sauer et al. highlight drug repurposing as a therapeutic approach in this context. Furthermore, both articles consider the patient perspective to explore in detail the challenges and shortcomings of current clinical trial design to target this early stage of metastasis.

Nevertheless, some of the questions posed by Paget more than a century ago remain unanswered: “What is it that decides what organs shall suffer in case of disseminated cancer?” (1). Hopefully, we are closer (just few steps away) to unravelling the complexity of the metastatic ecosystems. We invite the reader to enjoy this Research Topic containing current hot topics in the field of dormancy of disseminated tumour cells.

## Author contributions

All authors listed have made a substantial, direct, and intellectual contribution to the work and approved it for publication.
